# Supplementary material for: KCNA2 variants cause dilated cardiomyopathy, obesity and sleep apnea through RAC-ERK pathway
Source: EMBO Mol Med. 2026 Feb 24;18(4):1134–49. doi: 10.1038/s44321-026-00391-y (PMC13083917; doi:10.1038/s44321-026-00391-y)
Supplement: Supplementary file 1 — Appendix [file 44321_2026_391_MOESM1_ESM.pdf]

Appendix Figures: Contents

Page 2. Appendix Fig. S1: The P/LP/VUS variants in known DCM or obesity or sleep apnea-related genes

Page 3-4. Appendix Fig. S2: KCNA2 p.T184I display defective voltage-dependent currents and cell surface trafficking.

Page 5-6. Appendix Fig. S3: KCNA2 variants induce heart failure phenotype in cardiomyocytes.

Page 7-8. Appendix Fig. S4: KCNA2 p.T184K and p.R189W variants induce pathogenesis via RAC1 - ERK1/2 signalling.

Page 9-10. Appendix Fig. S5: RACi treatment rescues *KCNA2* variants induced heart failure phenotype in cardiomyocytes.

Page 11. Appendix Fig. S6: Simvastatin treatment reduced RAC1 activity in both KCNA2 p.T184K and p.R189W expressing cells.

Page 12-13. Appendix Fig. S7: KCNA2 p.T184I variant induces heart failure phenotype in cardiomyocytes via increased RAC1-ERK1/2 activation and is rescued by treatment with simvastatin.

Page 14-15. Appendix Fig. S8: Characterization of the KCNA2 p.T184K patient-derived iPSC line.

Page 16-17. Appendix Fig. S9: Characterization of the familial control iPSC line.

Page 18-19. Appendix Fig. S10: Cardiac organoids from KCNA2 p.T184K iPSC display heart failure phenotype.

Page 20. Appendix Fig. S11: Gene ontology (GO) pathway analysis in the treated KCNA2 p.T184K iPSC-CMs displaying lipid and cellular trafficking dysregulation.

Page 21-23. Appendix Fig. S12: Simvastatin rescues the DOSA phenotypes recapitulated in the KI *Drosophila* model.

Page 24-34. Appendix Table S1: Summary of statistical tests and p values.

Page 35-36. Appendix Table S2: List of primers used in the study.

.

Genes	Healthy individual P2	Patient P3	Patient P8	Associated disease
BAG3	None	None	None	DCM
DES	None	None	None	
FLNC	None	None	None	
LMNA	None	None	None	
MYH7	None	None	None	
PLN	None	None	None	
RBM20	None	None	None	
SCN5A	None	None	None	
TNNC1	None	None	None	
TNNT2	None	None	None	
TTN TV	None	None	None	
DSP	None	None	None	
ACTC1	None	None	None	
ACTN2	None	None	None	
JPH2	None	None	None	
NEXN	None	None	None	
TNNI3	None	None	None	
TPM1	None	None	None	
VCL	None	None	None	
NOX4	None	None	None	Obesity
HTRA1	None	None	None	
KLHL29	None	None	None	
TNF	None	None	None	Sleep Apnea
PTGER3	None	None	None	
LPAR1	None	None	None	

Appendix Fig. S1: The P/LP/VUS variants in known DCM or obesity or sleep apnea-related genes.

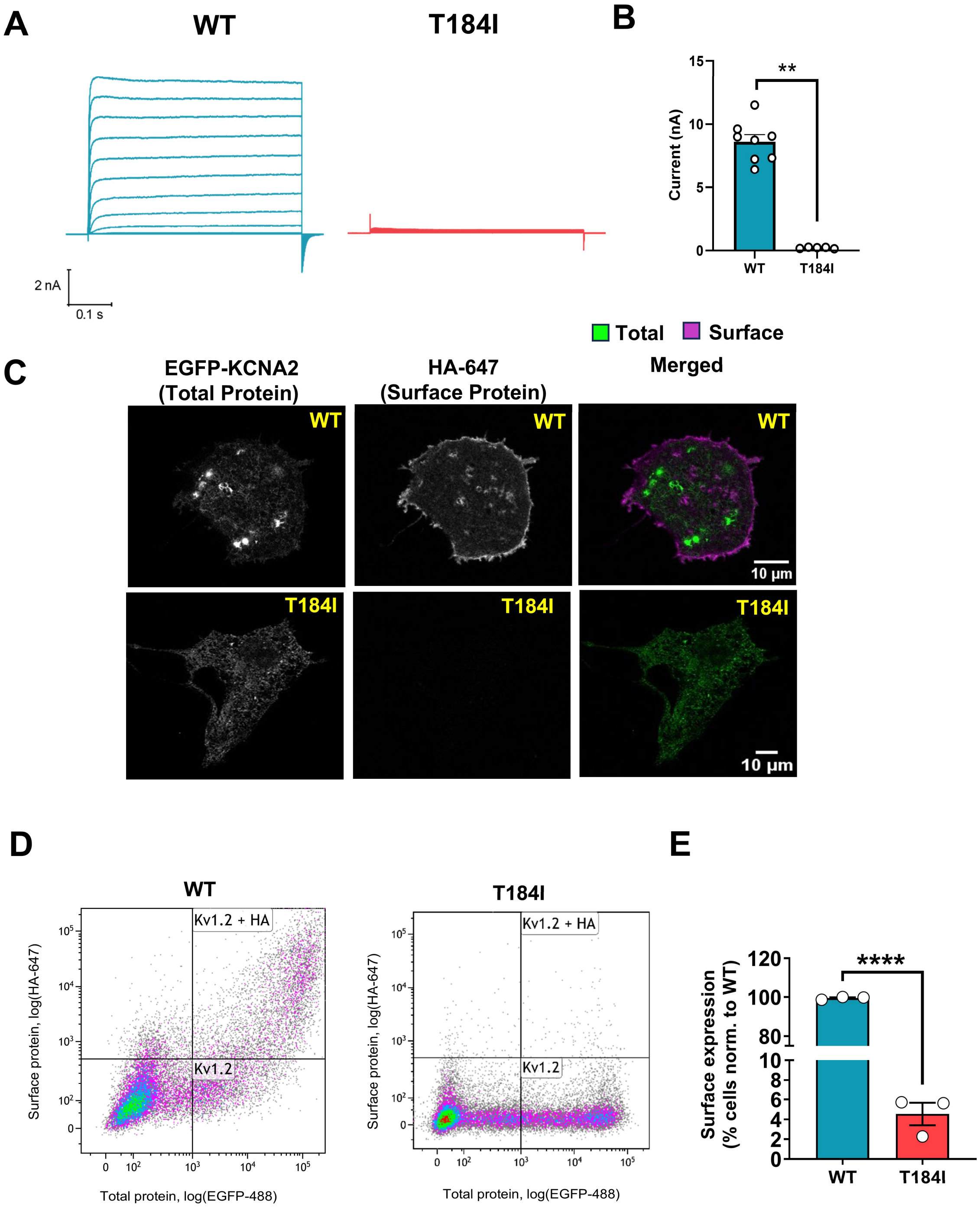

Appendix Fig. S2: KCNA2 p.T184I display defective voltage-dependent currents and cell surface trafficking. A) Whole-cell patch-clamp analysis of KCNA2 wild-type (light blue), T184I (red). Representative currents (nA) were elicited by step depolarization of HEK293T cells expressing the heterologous channels. The protocol comprised 13 depolarization pulses of 500ms duration every 10s from -60mV to +60mV. **B**) Total

currents (mean ± SEM) recorded from each channel at a potential of +60mV are presented. (WT n=8, T184I n=5) Significance was evaluated by unpaired t test, ****p<0.0001. **C**) Confocal micrographs of non-permeabilized COS7 cells overexpressing KCNA2 WT or p.T184I constructs. Scale bar= 10 μm. **D**) Flow cytometric analysis of live COS7 cells expressing *KCNA2* WT, p.T184K, and p.R189W. Plots representing cell density of log (EGFP) (total KCNA2) against log (HA) (KCNA2 on the cell surface). The vertical line separates the low- and high-EGFP cells, and the horizontal line separates the α-HA negative and positive cells (WT n=7066, T184I n=8631, KCNA2 positive live cells). **E)** The percentage of cells showing cell surface expression of KCNA2 (WT or variants) normalized to that of WT. The experiment was performed in triplicates (n=3). The values indicate the mean ± SEM. Significance was evaluated using unpaired t test ****p<0.000.1. The values are from biological replicates represented as means ±SEM.

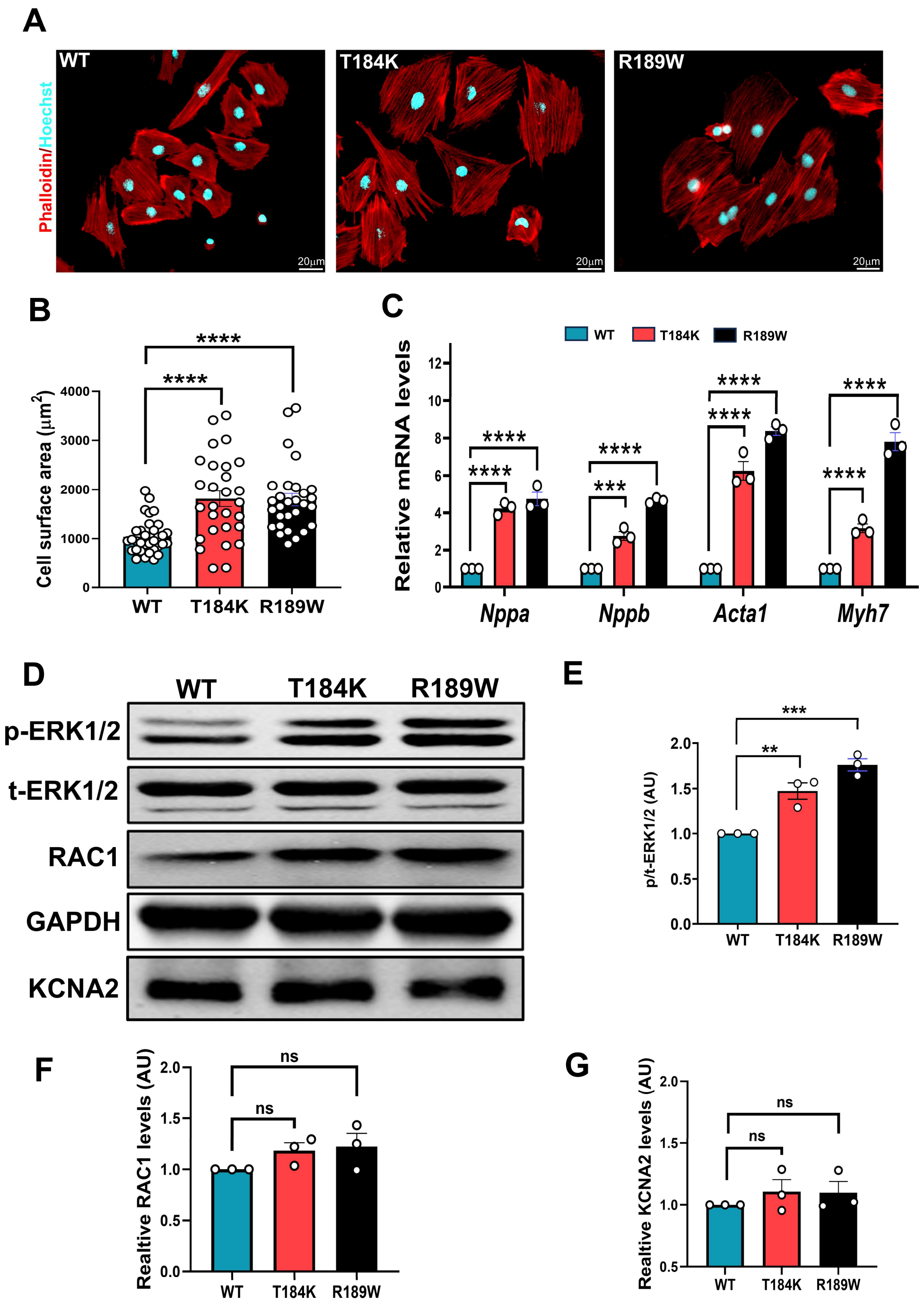

Appendix Fig. S3: ***KCNA2*** **variants induce heart failure phenotype in cardiomyocytes. A)** Representative images of H9c2 cardiomyocytes expressing WT p.T184K and p.R189W stained with Phalloidin (red) and Hoechst (cyan) scale bar=20µm. **B)** Cell surface area measurements of cells expressing WT (n=33 cells), p.T184K (n=28 cells) and p.R189W (n=29 cells). Significance was evaluated using Kruskal-Wallis One-way ANOVA test with post hoc Dunn's multiple comparisons test, ****$p<0.0001$. **C)** Quantitative real-time PCR analysis of heart failure markers *Nppa, Nppb, Myh7* and *Acta1*

in H9c2 cardiomyocytes expressing WT, p.T184K and p.R189W. The mRNA levels were normalised to *RNU6-1* and presented as relative expression levels compared with the level in the WT expressing cells. Each experiment was performed in triplicate (n=3 per group). The values are shown as mean±SEM. Significance was evaluated using Two-way ANOVA with post hoc Dunnett's multiple comparisons test, ***p<0.001, ****p<0.0001. D**)** Representative immunoblots of the indicated proteins from the whole cell lysates of H9c2 cardiomyocytes expressing WT, p.T184K and p.R189W. **E, F&G)** Densitometric analysis of the ratio of p-ERK1/2 to t-ERK1/2, RAC1 and KCNA2 normalized to the WT respectively (n=3 per group). Each experiment was performed in triplicate, and the values are shown as mean±SEM. Significance was evaluated using Ordinary one-way ANOVA with post hoc Dunnett's multiple comparisons, **p<0.01, ***p<0.001, ns not significant. The values are from biological replicates represented as means ±SEM.

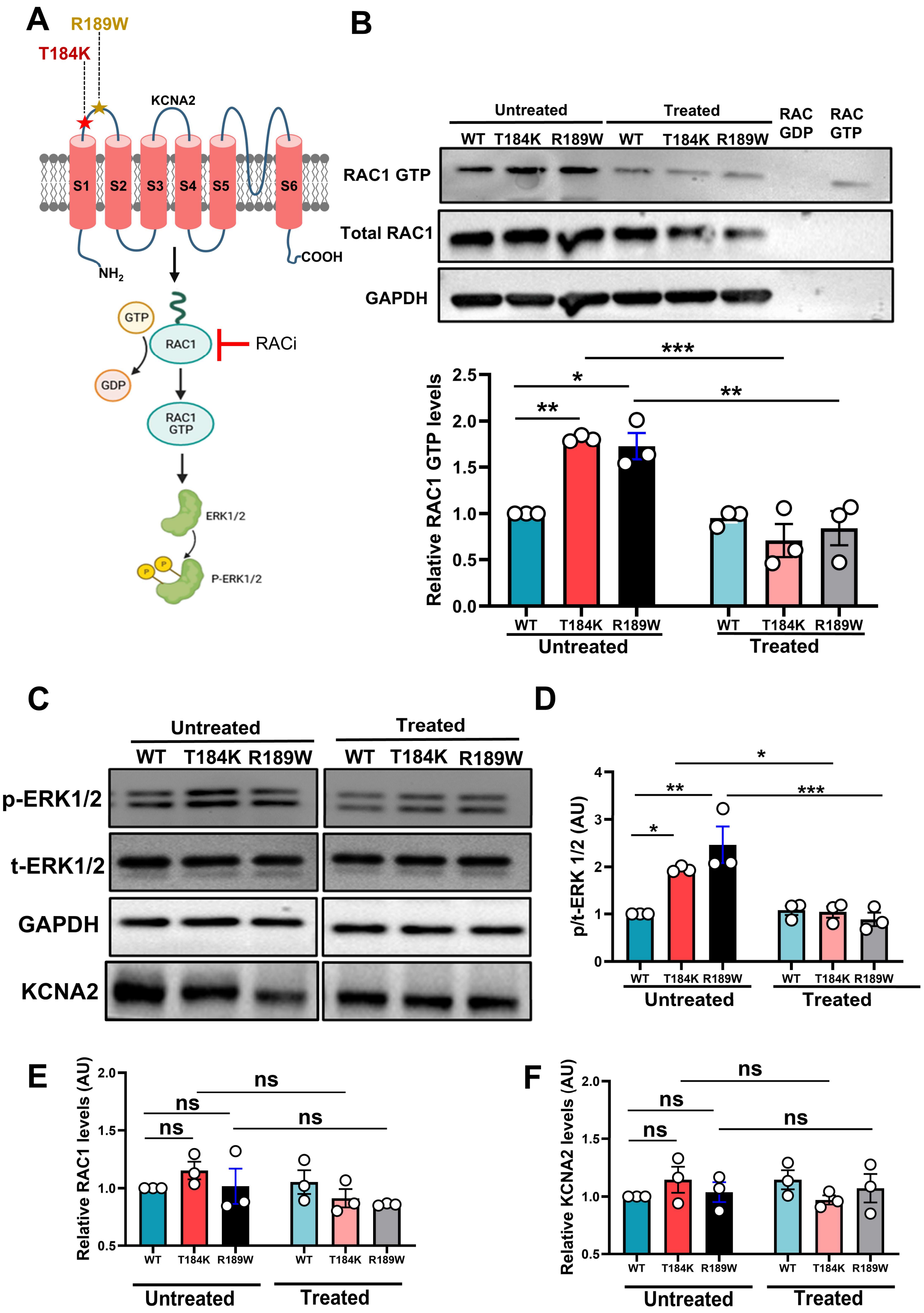

Appendix Fig. S4: KCNA2 p.T184K and p.R189W variants induce pathogenesis via RAC1 - ERK1/2 signalling. A) A schematic diagram of KCNA2 mediated ERK1/2 activation via RAC1 signaling. **B)** Immunoblots of RAC1 activity assay along with the total

lysate of H9c2 cardiomyocytes expressing WT, p.T184K and p.R189W in untreated and treated conditions (EHT1864 10μM for 2 h post 46 h of transfection) and densitometric analysis of RAC1 GTP normalized to the WT untreated. The untreated KCNA2 variant expressing cells were compared with the untreated WT or respective treated groups as indicated. Each experiment was performed in triplicate(n=3), and the values are shown as means ±SEM. Significance was evaluated using Two-way ANOVA with post hoc Tukey's multiple comparisons test, *p<0.05,**p<0.01,***p<0.001.**C)** Representative immunoblots of the indicated proteins from the whole cell lysate of KCNA2 p.T184K and p.R189W expressing H9c2 cells. **D,E&F**) Densitometric analysis of the ratio of p-ERK to total ERK1/2, RAC1 and KCNA2 levels normalized to that of WT respectively. The untreated KCNA2 variant expressing cells were compared with the untreated WT or respective treated groups as indicated. Each experiment was performed in triplicate (n=3), and the values are shown as means ±SEM. Significance was evaluated using Two-way ANOVA with post hoc Tukey's multiple comparisons test, respectively,*p<0.05,**p<0.01,***p<0.001. The values are from biological replicates represented as means ±SEM.

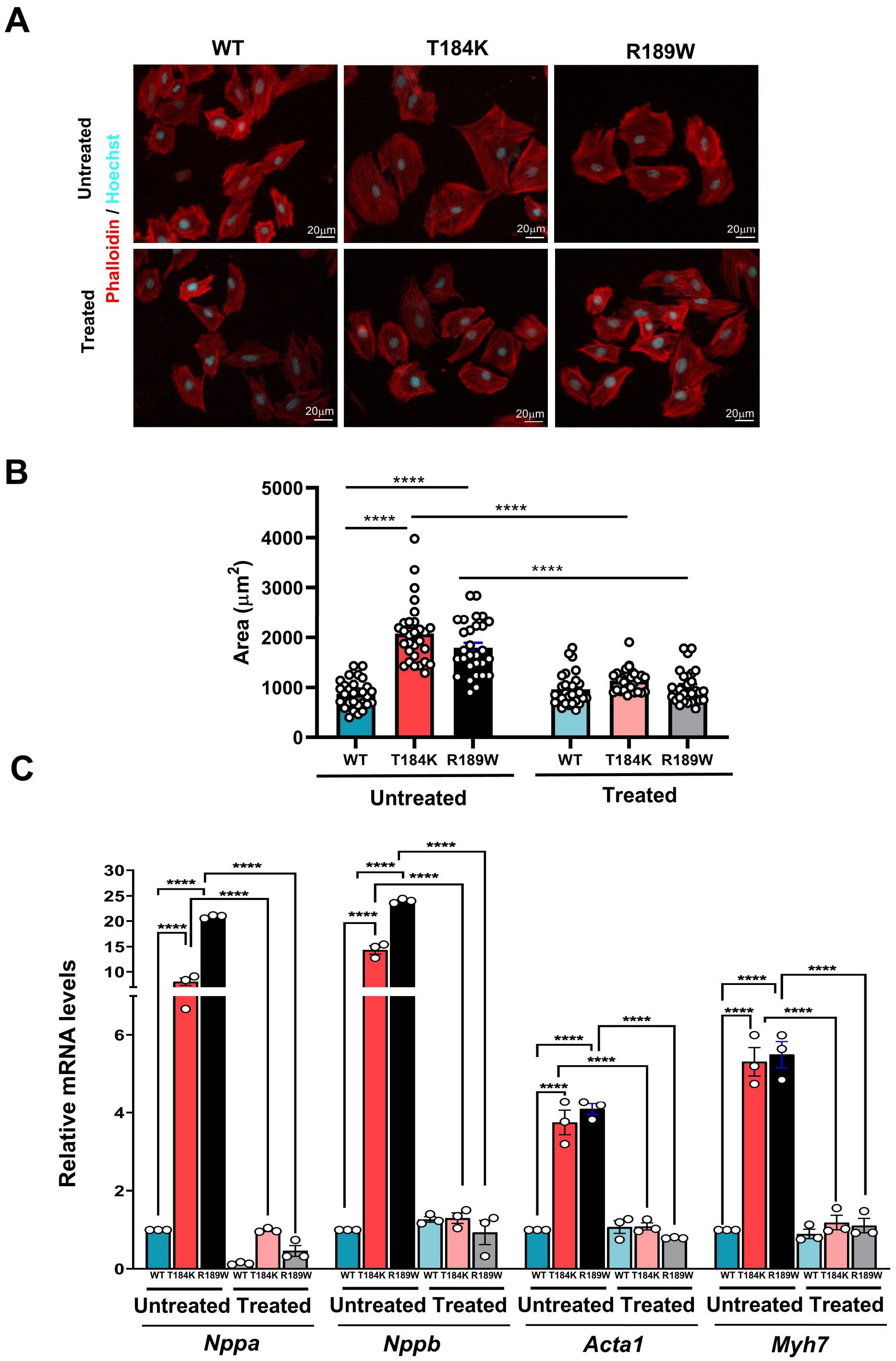

Appendix Fig. S5: RACi treatment rescues KCNA2 variants induced heart failure phenotype in cardiomyocytes. A) Representative images of H9c2 cardiomyocytes expressing WT, p.T184K and p.R189W in untreated and treated conditions (EHT1864 10μM for 2 hours post 48 hrs of transfection) stained with Phalloidin (red) and Hoechst (cyan) Scale bar =20μm. **B)** Cell surface area measurements of cells expressing WT, p.T184K and p.R189W in untreated and treated conditions (n=29 cells per group). The

untreated KCNA2 variant expressing cells were compared with the untreated WT or respective treated groups as indicated. The values are shown as mean±SEM. Significance was evaluated using Two-way ANOVA with post hoc Tukey's multiple comparisons test, ****p<0.0001. **C)** Quantitative real-time PCR analysis of heart failure markers *Nppa, Nppb, Myh7* and *Acta1* in H9c2 cardiomyocytes expressing WT, p.T184K and p.R189W in treated and untreated conditions. The mRNA levels were normalised to 18s rRNA and presented as relative expression levels of untreated WT cells. The untreated KCNA2 variant expressing cells were compared with the untreated WT or respective treated groups as indicated. Values of each experiment performed in triplicate (n=3). Each experiment was performed in triplicate, and the values are shown as mean±SEM. Significance was evaluated using the Two-way ANOVA, with post hoc Tukey's multiple comparisons test ****p<0.0001. The values are from biological replicates represented as means ±SEM.

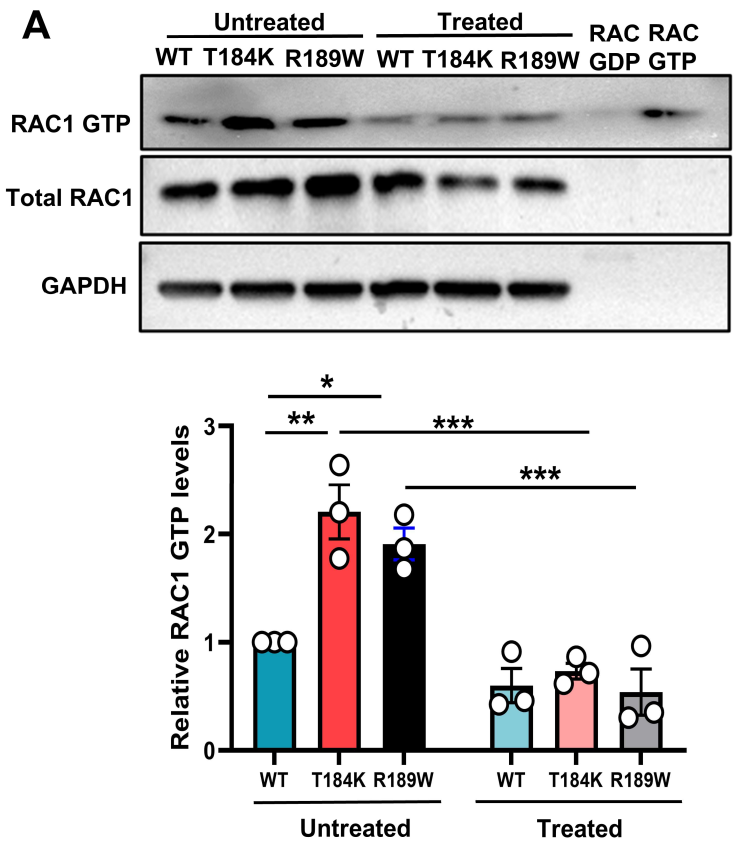

Appendix Fig. S6: Simvastatin treatment reduced RAC1 activity in both KCNA2 p.T184K and p.R189W expressing cells. A) Representative Immunoblots for RAC1 GTP (RAC1 activity assay) and total proteins (Total RAC1 and GAPDH) in the whole-cell lysate of the H9C2 cardiomyocytes expressing WT, p.T184K and p.R189W in untreated and treated (simvastatin 12μM for 24 h) conditions. Densitometric analysis of RAC1 levels normalized to that of the WT. The untreated KCNA2 variant expressing cells were compared with the untreated WT or respective treated groups as indicated. Each experiment was performed in triplicate(n=3) and the values are shown as means ±SEM. Significance was evaluated using Two-way ANOVA with post hoc Tukey's multiple comparisons test, *p<0.05,**p<0.01,***p<0.001. The values are from biological replicates represented as means ±SEM.

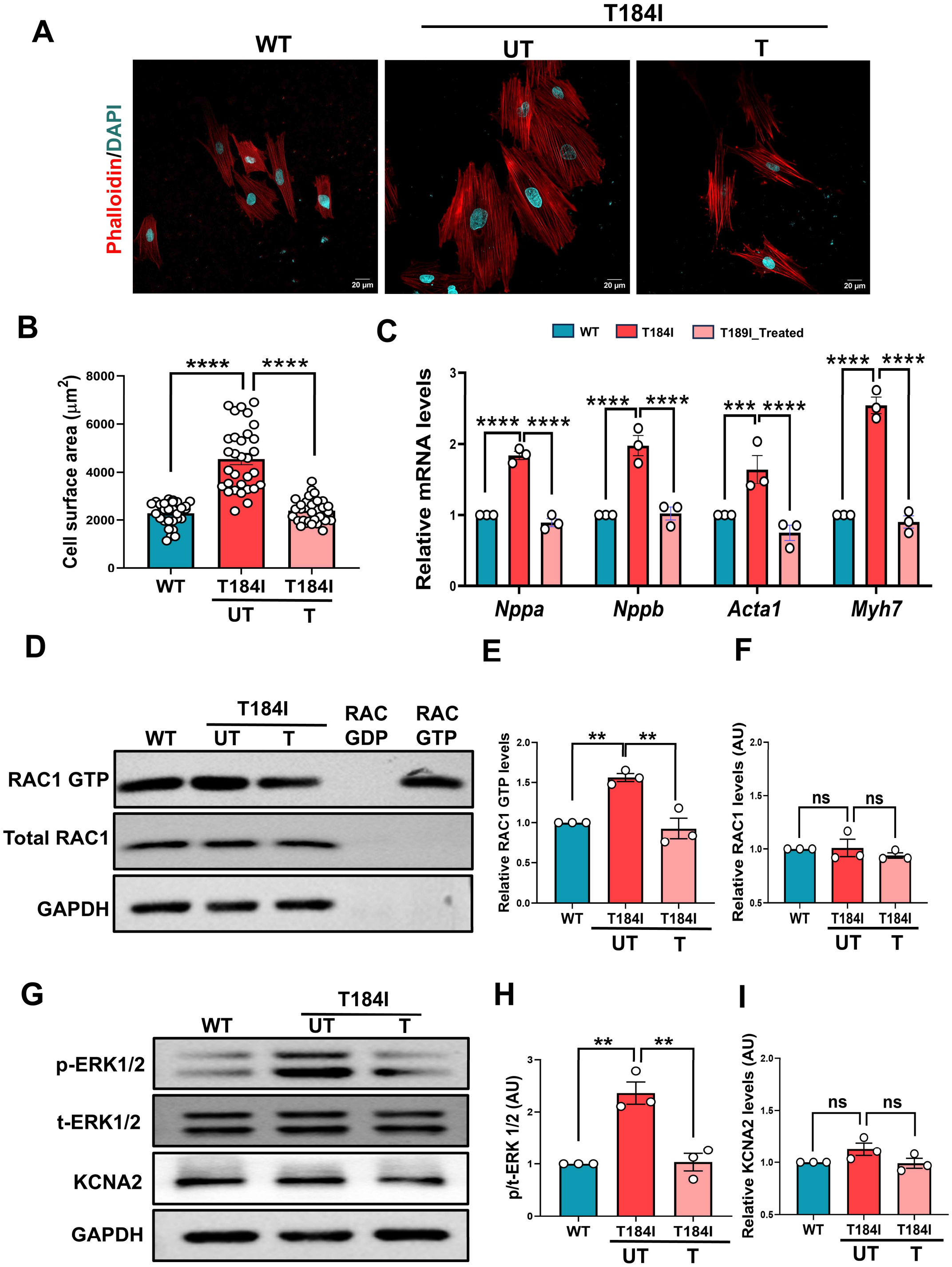

Appendix Fig. S7: KCNA2 p.T184I variant induces heart failure phenotype in cardiomyocytes via increased RAC1-ERK1/2 activation and is rescued by treatment with simvastatin. A) Representative images of H9c2 cardiomyocytes expressing WT and p.T184I (UT: untreated and T: treated) stained with Phalloidin (red) and Hoechst (cyan) scale bar=20μm. **B**) Cell surface area measurements of cells expressing WT and p.T184I

(UT and T) 31 cells in each group. Significance was evaluated using One-way ANOVA with post hoc Tukey's multiple comparisons test, ****p<0.0001. **C**) Quantitative real-time PCR analysis of heart failure markers *Nppa, Nppb, Myh7* and *Acta1* in H9c2 cardiomyocytes expressing WT, and p.T184I(untreated and treated). The mRNA levels were normalised to RNU6-1 and presented as relative expression levels compared with the level in the WT expressing cells. Each experiment was performed in triplicate (n=3). The values are shown as mean±SEM. Significance was evaluated using Two-way with post hoc Tukey's multiple comparisons test,****p<0.0001. **D**) Immunoblots of RAC1 activity assay along with the total lysate of H9c2 cardiomyocytes expressing WT and p.T184I. **E&F**) Densitometric analysis of RAC1 GTP normalized to the WT and the total RAC1 levels respectively. Each experiment was performed in triplicate(n=3), and the values are shown as means ±SEM. Significance was evaluated using One-way ANOVA with post hoc Tukey's multiple comparisons test, **p<0.01, ns not significant. **G**) Representative immunoblots of the indicated proteins from the whole cell lysates of H9c2 cardiomyocytes expressing WT and p.T184I. **H&I**) Densitometric analysis of the ratio of p-ERK1/2 to t-ERK1/2 and KCNA2 levels normalized to the WT (n=3). Each experiment was performed in triplicate, and the values are shown as mean±SEM. Significance was evaluated using One-way ANOVA with post hoc Tukey's multiple comparisons test, **p<0.01, ns not significant. The values are from biological replicates represented as means ±SEM.

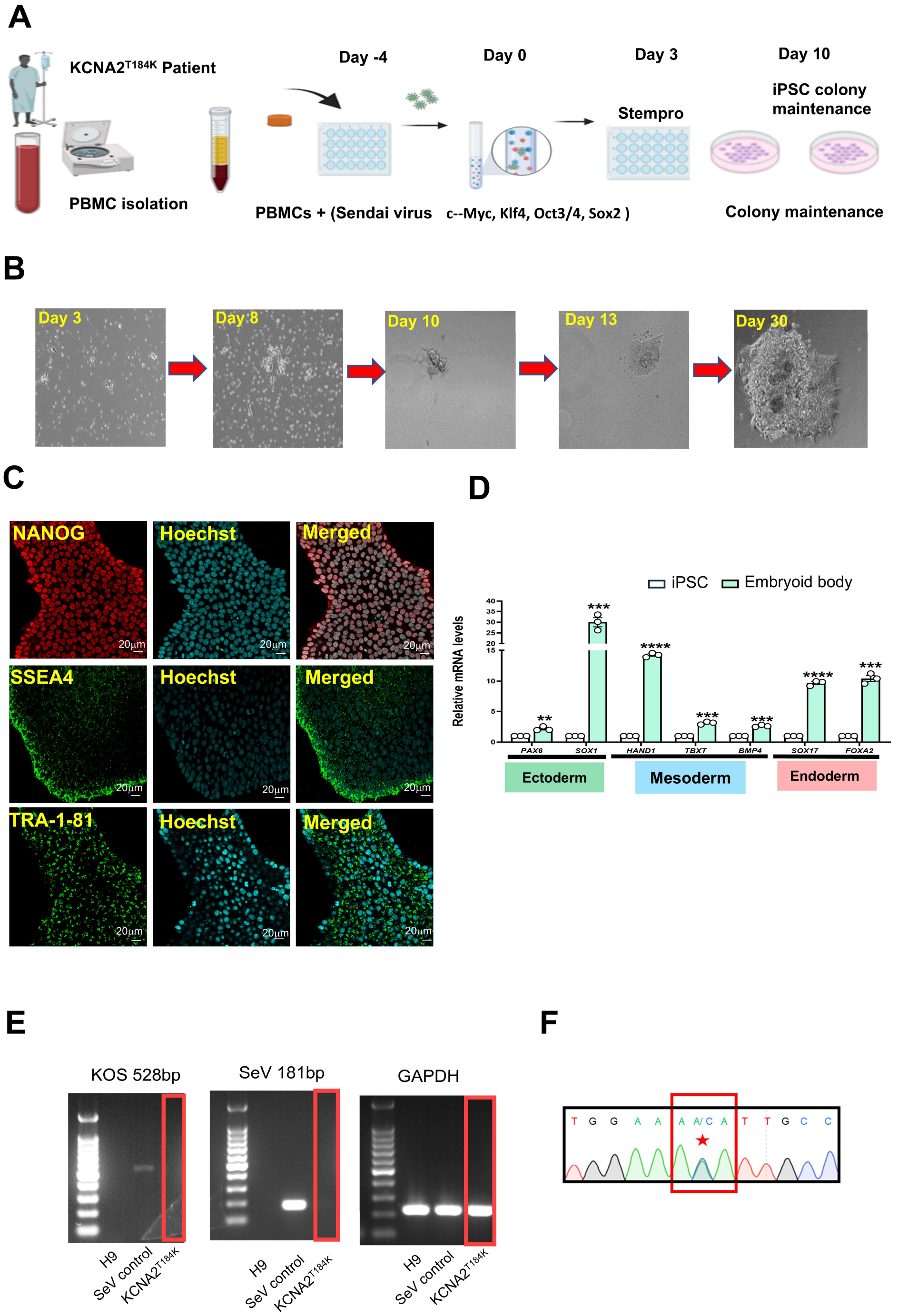

Appendix Fig. S8: Characterization of the KCNA2 p.T184K patient-derived iPSC line. A) Schematic diagram for generation of iPSC from peripheral blood mononuclear cells (PBMCs) of KCNA2 p.T184K patient. **B)** Representative bright field images at different time points of iPSC colony generation. **C)** Confocal micrographs of iPSCs for stemness markers NANOG (red), SSEA4(green), TRA-1-81(green), Hoechst (cyan). Scale bar =20μm **D)** Quantitative real time PCR for the expression of tri-germ layer

markers in the patient-specific iPSC-derived embryoid bodies relative to patient-derived iPSC. mRNA levels were normalised to *18s rRNA* as housekeeping gene and presented as relative expression levels compared with the level in patient-derived iPSC cells. The values represented are means ± SEM. n= 3 repeats. Significance was calculated between iPSC and embryoid body transcripts using multiple t-tests with post hoc Holm-Sidak method, ***p<0.001,****p<0.0001. The values are from biological replicates represented as means ±SEM. **E)** Reverse Transcriptase PCR to validate the absence of the Sendai virus. H9 embryonic stem cell line was used as the negative control and Sev Control was used as a positive control **F**) Sequence confirmation of the KCNA2 p.T184K mutation in the patient (P3) derived iPSC line.

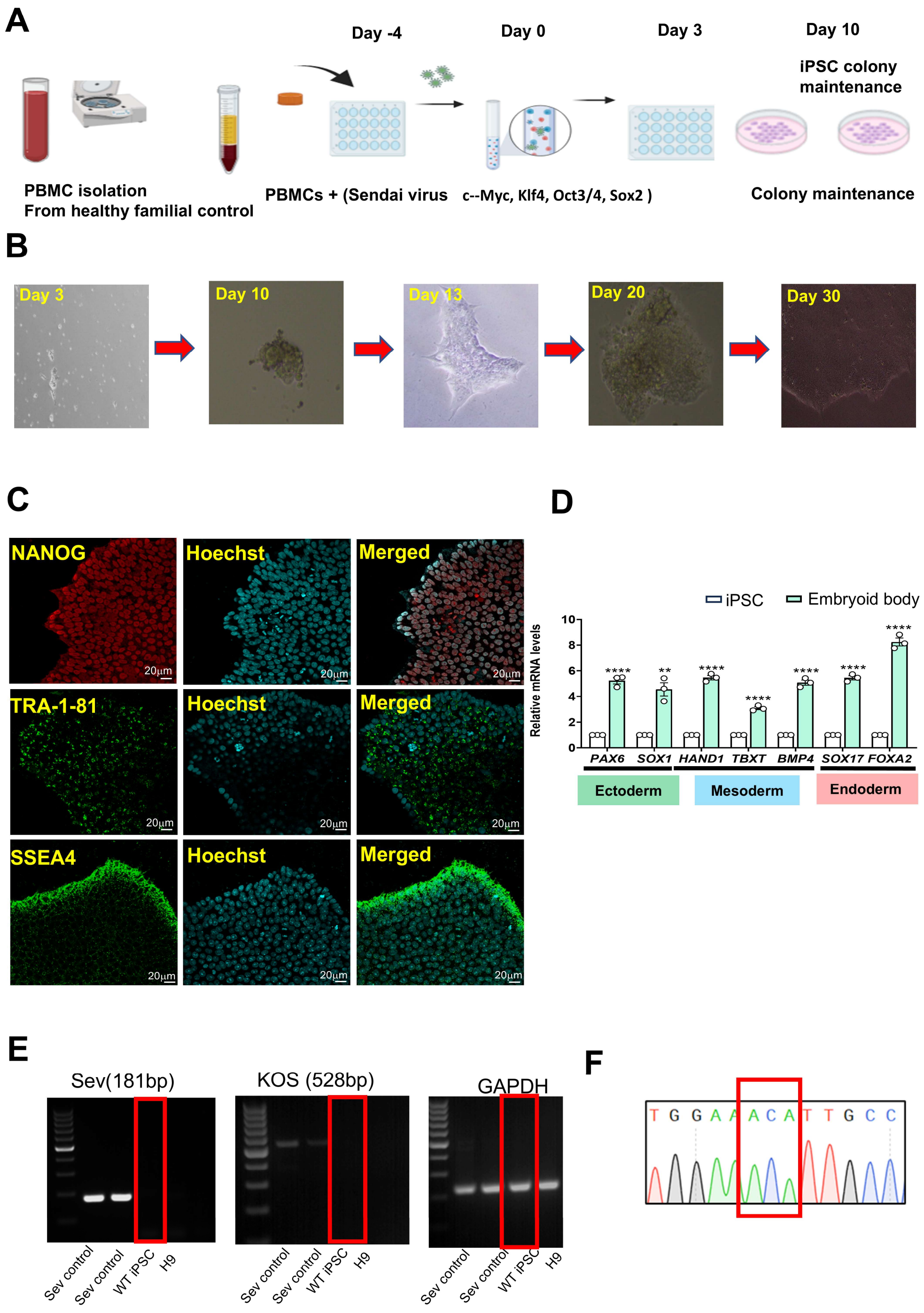

Appendix Fig. S9: Characterization of the familial control iPSC line. A) Schematic diagram for generation of iPSC from peripheral blood mononuclear cells (PBMCs) of the familial control(P2). **B)** Representative bright field images at different time points of iPSC colony generation. **C)** Confocal micrographs of iPSCs for stemness markers NANOG (red), SSEA4 (green), TRA-1-81 (green), Hoechst (cyan) Scale bar =20μm. **D)**

Quantitative real time PCR for the expression of tri-germ layer markers in the control-specific iPSC-derived embryoid bodies relative to control-derived iPSC. mRNA levels were normalised to *18s rRNA* as housekeeping gene and presented as relative expression levels compared with the level in control-derived iPSC cells. The values represented are means ± SEM. n=3 repeats. Significance was calculated between iPSC and embryoid body transcripts using multiple t-tests with post hoc Holm-Sidak method, ***p<0.001,****p<0.0001. The values are from biological replicates represented as means ±SEM. **E)** Reverse Transcriptase PCR to validate the absence of the Sendai virus. H9 embryonic stem cell line was used as the negative control and Sev Control was used as the positive control **F)** Sequence confirmation of the KCNA2 WT in the familial control (P2) derived iPSC line.

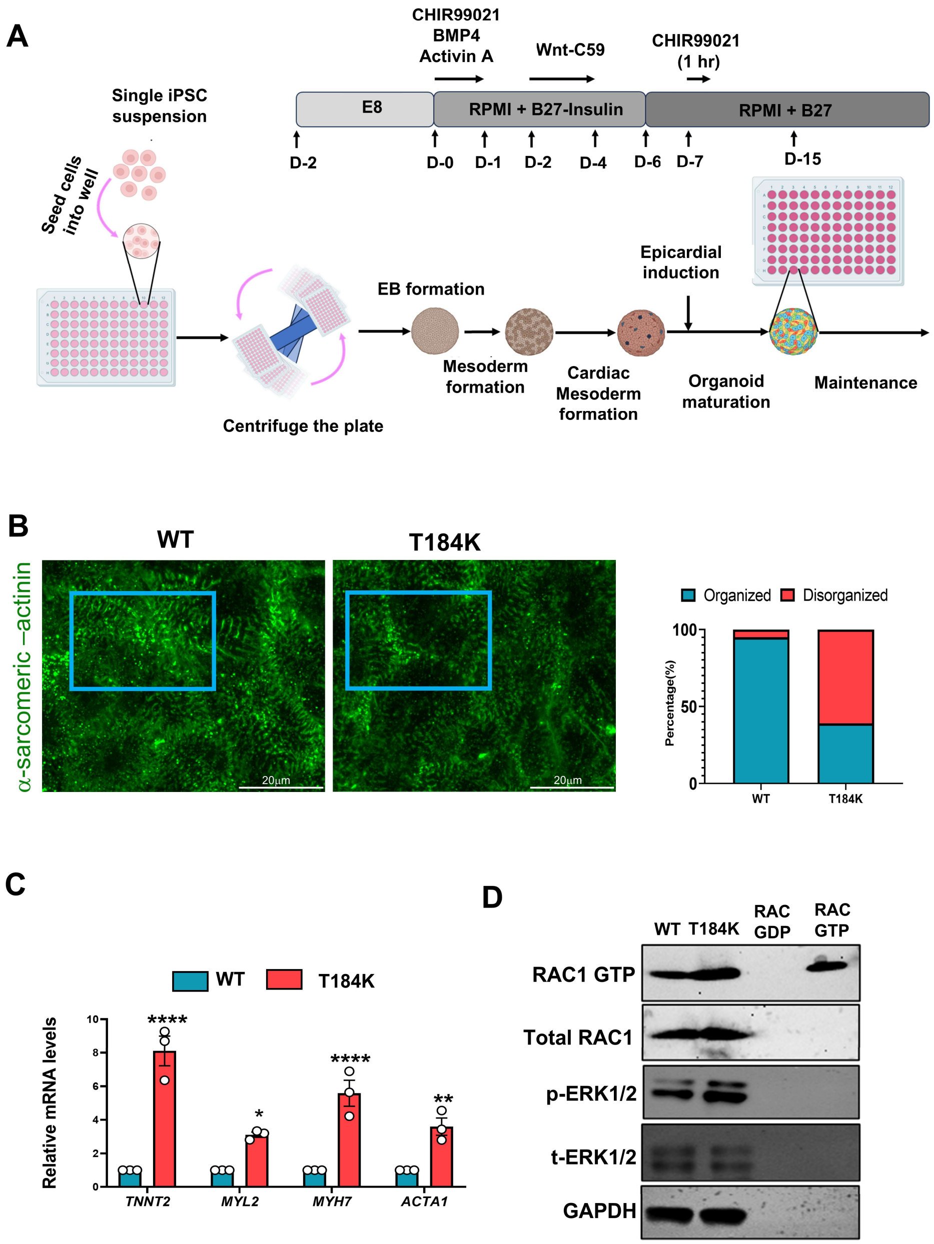

Appendix Fig. S10: Cardiac organoids from KCNA2 p.T184K iPSC display heart failure phenotype. A) Schematic diagram for generation of cardiac organoids. **B**) Confocal micrographs of cardiac organoids stained with α-sarcomeric actinin(green). Scale bar =20μm. **C)** Quantitative real time PCR for heart failure related genes. mRNA levels were normalised to those of WT and 18s rRNA was used as the internal control. The experiment was performed in triplicate, and the values are represented as means ±

SEM. Statistical test carried out using Two-way ANOVA with post hoc Sidak's multiple comparisons test, *$p<0.05$,**$p<0.01$ and ****$p<0.0001$. The values are from biological replicates represented as means ±SEM. **D)** Immunoblots of RAC1 activity assay in cardiac organoids along with the total lysate blot with the indicated proteins.

A

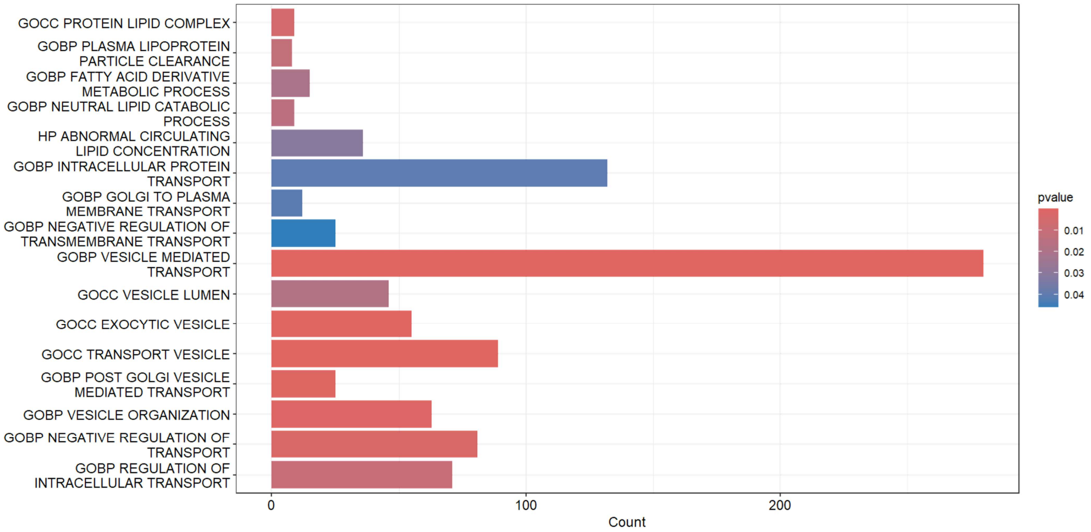

Appendix Fig. S11: Gene ontology (GO) pathway analysis in the treated KCNA2 p.T184K iPSC-CMs displaying lipid and cellular trafficking dysregulation. A) Bar plot representing significant pathway enrichment in untreated and treated (p.T184K) iPSC-CMs.

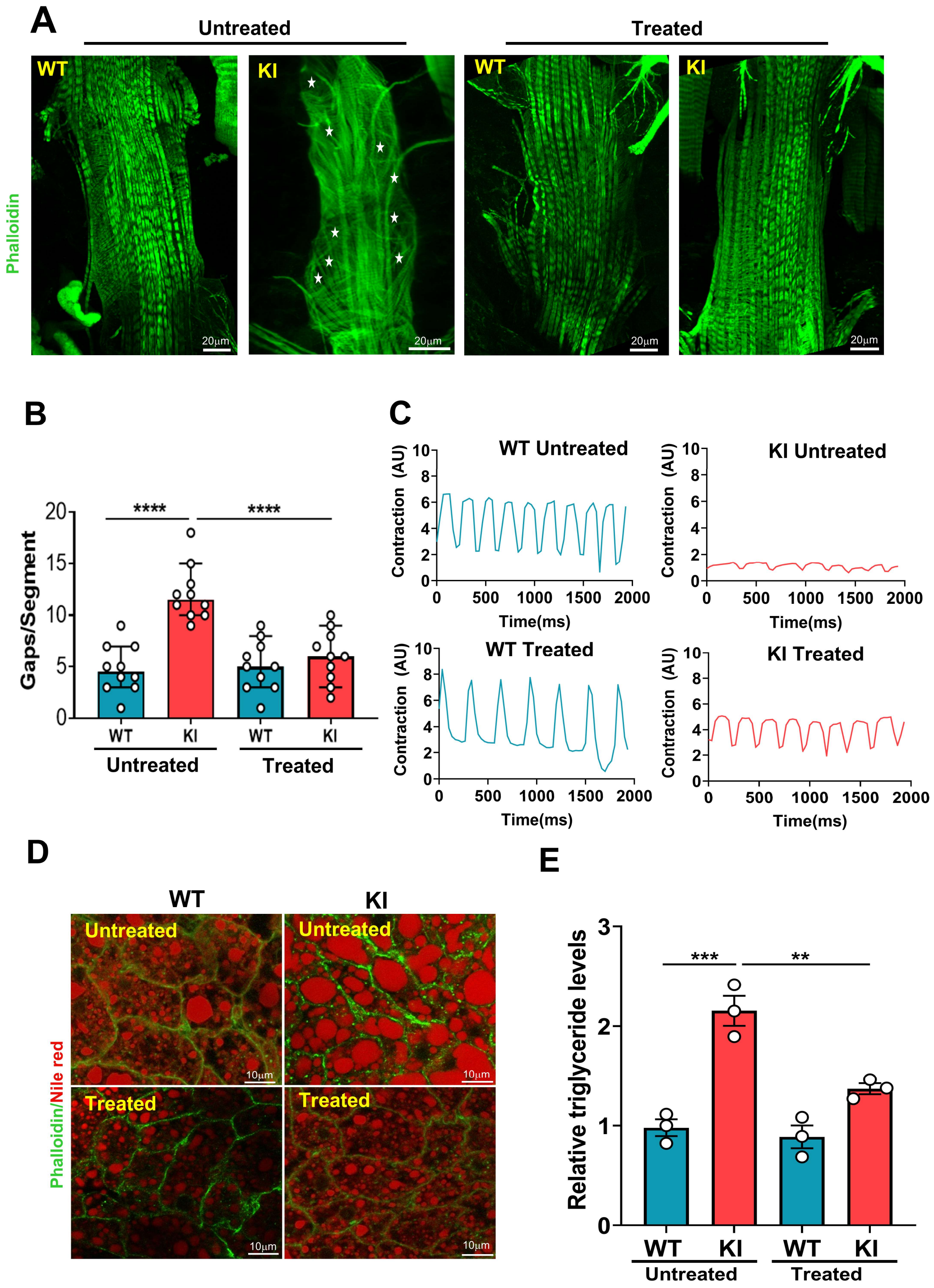
A
Untreated
Treated
WT
KI
WT
KI
Phalloidin
20µm
B
Gaps/Segment
20
15
10
5
0
****
****
WT
KI
WT
KI
Untreated
Treated
C
WT Untreated
KI Untreated
WT Treated
KI Treated
Contraction (AU)
Time(ms)
0
500
1000
1500
2000
D
WT
KI
Untreated
Treated
Phalloidin/Nile red
10µm
E
Relative triglyceride levels
3
2
1
0
***
**
WT
KI
WT
KI
Untreated
Treated

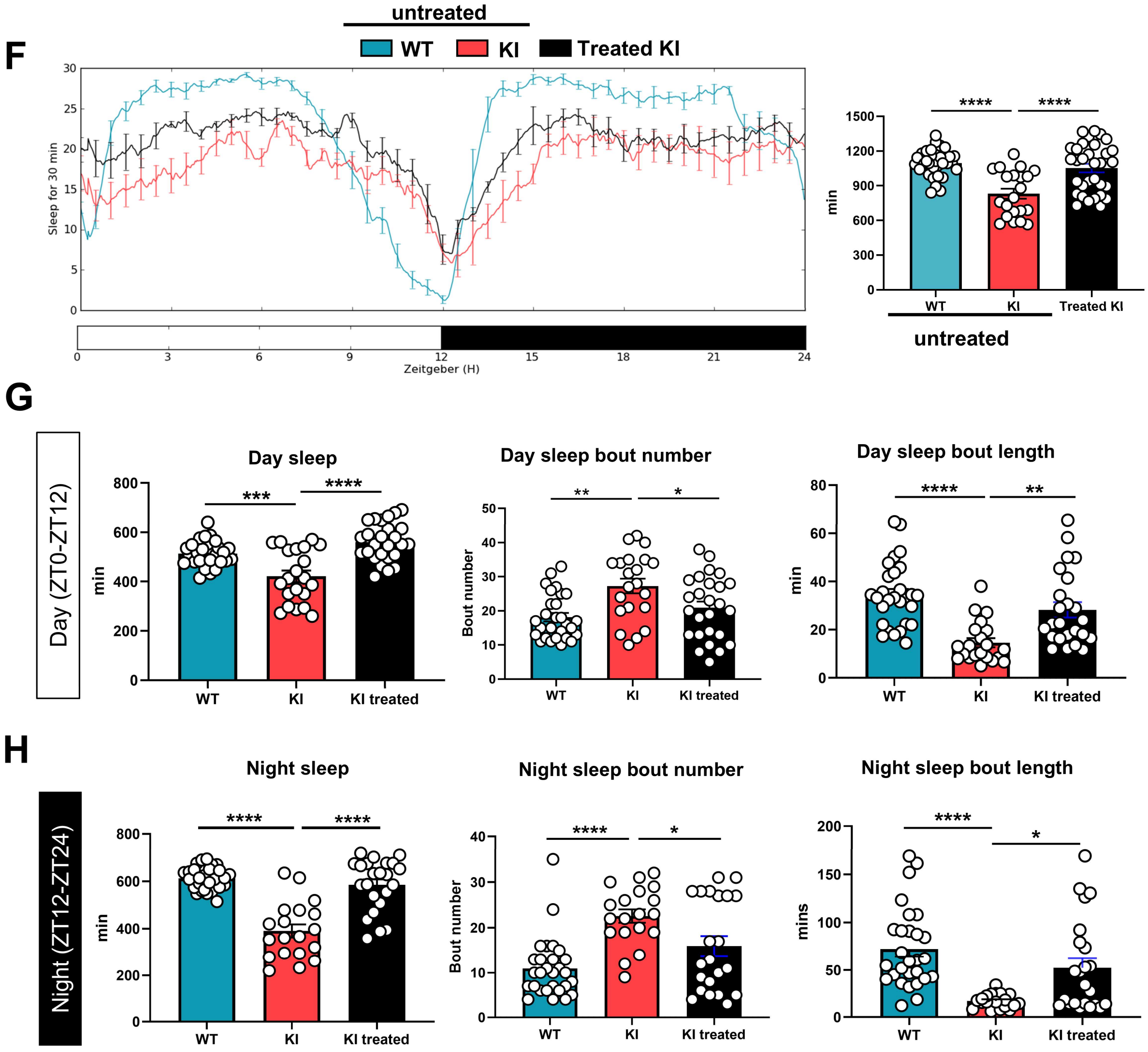

Appendix Fig. S12: Simvastatin rescues the DOSA phenotypes recapitulated in the KI *Drosophila* model. A) Heart tubes of the WT and KI *Drosophila* (untreated and treated) were stained with phalloidin(green) with the stars(white) highlighting the gaps in the segment. Scale bar =20μm. **B**) quantification of gaps per segments (n=10 flies per group). The untreated KI *Drosophila* were compared with the untreated WT or treated KI as indicated. Statistical test carried out using Two-way ANOVA with Sidak's multiple comparisons test, ****p<0.0001. **C)** The heart tube contraction amplitude traces from live semi-intact heart tubes of the WT and KI in untreated and treated and their quantifications. **D**) Confocal micrographs of the abdominal fat bodies from WT and KI stained for Nile red, with the fat body (red) and Phalloidin(green). Scale bar =10μm. **E**) Relative triglyceride levels of the WT and KI (untreated and treated) *Drosophila* normalised to the WT using

triglyceride assay (n=3). The untreated KI *Drosophila* were compared with the untreated WT or treated KI as indicated. The values are represented as means ± SEM. Statistical test carried out using one-way ANOVA with post hoc Tukey's multiple comparisons test,**p<0.01 ***p<0.001.The values are from biological replicates represented as means ±SEM. **F**) Sleep traces of WT, KI (untreated and treated) *Drosophila* in a 12-h dark and light cycle(left).Total amount of sleep in a day (ZT-24h) (right) (WT n=32, KI n=21, KI treated n=32 flies per group). The untreated KI *Drosophila* were compared with the untreated WT or treated KI as indicated. The values represented are means ± SEM Significance was evaluated using ordinary one-way ANOVA, with post hoc Tukey's multiple comparisons test, ****p<0.0001. **G**) Day time sleep quantifications (ZT0-12 h) (WT n=28, KI n=21, KI treated n=26 flies per group). **H**) Night time sleep quantifications (ZT-12-24h) (WT n=28, KI n=19, KI treated n=23 flies per group). The values represented are means ± SEM. Significance was evaluated using ordinary one-way ANOVA, with post hoc Tukey's multiple comparisons test, *p<0.05 **p<0.01 ***p<0.001****p<0.0001, ns: non-significant.

Appendix Table S1: Summary of statistical tests and p values

Figure #	Statistical method	Multiple comparison	Groups	P value	summary
**Figure 2**					
2B	One-way ANOVA	Dunnett's multiple comparisons test	WT vs T184K	2.2E-17	****
			WT vs. R189W	1.7E-16	****
2F	One-way ANOVA	Dunnett's multiple comparisons test	WT vs T184K	2.5E-10	****
			WT vs R189W	3.2E-10	****
**Figure 3**					
3B	Two-way ANOVA	Tukey's multiple comparisons test	***NPPA*** WT vs T184K untreated	0.0041	**
			NPPA T184K untreated vs. T184K treated	0.0050	**
			ACTA1 WT vs T184K untreated	1.8E-9	****
			ACTA1 T184K untreated vs. T184K treated	3.9E-9	****
			MYH6 WT vs T184K untreated	0.0099	**
			MYH6 T184K untreated vs. T184K treated	0.0052	**
**Figure 4**					
4A	Unpaired t test (Two tailed)	N/A	WT vs KI	2.0E-6	****

4B(Contraction Amplitude)	Unpaired t test (Two tailed)	N/A	WT vs KI	5.7E-5	****
4B(Contraction Velocity)	Unpaired t test (Two tailed)	N/A	WT vs KI	1.5E-5	****
4E	Unpaired t test (Two tailed)	N/A	WT vs KI	0.0002	***
**Appendix figures**					
**Appendix Figure S2**					
S2B	Unpaired t test(Two tailed)	N/A	WT vs T184I	4.4E-7	****
S2E	Unpaired t test(Two tailed)	N/A	WT vs T184I	2.7E-9	****
**Appendix Figure S3**					
S3B	Kruskal-Wallis One-way ANOVA test	Dunn's multiple comparisons test	WT vs. T184K	3.9E-5	****
			WT vs. R189W	5.0E-6	****
S3C	Two-way ANOVA	Dunnett's multiple comparisons test	***Nppa*** WT vs. T184K	1.2E-08	****
			Nppa WT vs. R189W	2.9E-10	****
			Nppb WT vs. T184K	1.5E-4	***
			Nppb WT vs. R189W	4.2E-09	****

			Acta1 WT vs. T184K	1.2E-12	****
			Acta1 WT vs. R189W	6.8E-16	****
			Myh7 WT vs. T184K	4.3E-06	****
			Myh7 WT vs. R189W	1.0E-12	****
S3E	One-way ANOVA	Dunnett's multiple comparisons test	WT vs T184K	2.1E-14	**
			WT vs. R189W	0.0003	***
S3F	One-way ANOVA	Dunnett's multiple comparisons test	WT vs T184K	0.2952	ns
			WT vs. R189W	0.1910	ns
S3G	One-way ANOVA	Dunnett's multiple comparisons test	WT vs T184K	0.5439	ns
			WT vs. R189W	0.5956	ns
**Appendix Figure S4**					
S4B	Two-way ANOVA	Tukey's multiple comparisons test	WT Untreated vs. T184K Untreated	0.0057	**
			WT Untreated vs. R189W Untreated	0.0123	*
			T184K Untreated vs. T184K Treated	0.0004	***
			R189W Untreated vs. R189W Treated	0.0028	**

S4D	Two-way ANOVA	Tukey's multiple comparisons test	WT Untreated vs. T184K Untreated	0.0270	*
			WT Untreated vs. R189W Untreated	0.0010	**
			T184K Untreated vs. T184K Treated	0.0362	*
			R189W Untreated vs. R189W Treated	0.0005	***
S4E	Two-way ANOVA	Tukey's multiple comparisons test	WT Untreated vs. T184K Untreated	0.8097	ns
			WT Untreated vs. R189W Untreated	>0.9999	ns
			T184K Untreated vs. T184K Treated	0.4266	ns
			R189W Untreated vs. R189W Treated	0.7966	ns
S4F	Two-way ANOVA	Tukey's multiple comparisons test	WT Untreated vs. T184K Untreated	0.8248	ns
			WT Untreated vs. R189W Untreated	0.9994	ns
			T184K Untreated vs. T184K Treated	0.6989	ns

			R189W Untreated vs. R189W Treated	0.9997	ns
**Appendix Figure S5**					
S5A					
	Two-way ANOVA	Tukey's multiple comparisons test	WT Untreated vs. T184K Untreated	2.4E-28	****
			T184K Untreated vs. T184K Treated	4.0E-14	****
			WT Untreated vs. R189W Untreated	7.7E-14	****
			R189W Untreated vs. R189W Treated	1.4E-11	****
S5C	Two-way ANOVA	Tukey's multiple comparisons test	***Nppa*** WT Untreated vs. T184K Untreated	2.6E-15	****
			Nppa T184K Untreated vs. T184K Treated	2.6E-15	****
			Nppa WT Untreated vs. R189W Untreated	2.6E-15	****
			Nppa R189W Untreated vs. R189W Treated	2.6E-15	****

			Nppb WT Untreated vs. T184K Untreated	2.6E-15	****
			Nppb T184K Untreated vs. T184K Treated	2.6E-15	****
			Nppb WT Untreated vs. R189W Untreated	2.6E-15	****
			Nppb R189W Untreated vs. R189W Treated	2.6E-15	****
			Acta1 WT Untreated vs. T184K Untreated	2.0E-07	****
			Acta1 T184K Untreated vs. T184K Treated	4.4E-07	****
			Acta1 WT Untreated vs. R189W Untreated	9.9E-09	****
			Acta1 R189W Untreated vs. R189W Treated	1.6E-09	****
			Myh7 WT Untreated vs. T184K Untreated	3.9E-13	****

			Myh7 T184K Untreated vs. T184K Treated	1.7E-12	****
			Myh7 WT Untreated vs. R189W Untreated	9.7E-14	****
			Myh7 R189W Untreated vs. R189W Treated	2.2E-13	****
**Appendix Figure S6**					
S6A	Two-way ANOVA	Tukey's multiple comparisons test	WT Untreated vs. T184K Untreated	0.0022	**
			WT Untreated vs. R189W Untreated	0.0183	*
			T184K Untreated vs. T184K Treated	0.0004	***
			R189W Untreated vs. R189W Treated	0.0007	***
**Appendix Figure S7**					
S7B	One-way ANOVA	Tukey's multiple comparisons test	WT vs T184I	4.3E−10	****
			T184I vs T184I_treated	4.3E−10	****
S7C	Two-way ANOVA	Sidak's multiple	***Nppa*** WT vs T184I	2.5E−10	****

		comparisons test			
			Nppa T184I vs T184I_treated	1.07E−12	****
			Nppb WT vs T184I	1.9E-13	****
			Nppb T184I vs T184I_treated	6.7E-13	****
			Acta1 WT vs T184I	1.3E-6	****
			Acta1 T184I vs T184I_treated	1.9E-11	****
			Myh7 WT vs T184I		****
			Myh7 T184I vs T184I_treated		****
S7E	One-way ANOVA	Tukey's multiple comparisons test	WT vs T184I	2.06E-3	**
			T184I vs T184I_treated	2.3E-3	**
S7F	One-way ANOVA	Tukey's multiple comparisons test	WT vs T184I	0.1921	ns
			T184I vs T184I_treated	0.1575	ns
S7H	One-way ANOVA	Tukey's multiple comparisons test	WT vs T184I	0.0063	**
			T184I vs T184I_treated	0.0033	**
S7I	One-way ANOVA	Tukey's multiple comparisons test	WT vs T184I	0.9902	ns

			T184I vs T184I_treated	0.5946	ns
**Appendix Figure S8**					
S8D	Multiple t-tests with post hoc Holm-Sidak method	N/A	***PAX6*** iPSC vs EB	0.0045	**
			SOX1 iPSC vs EB	3.3E-4	***
			HAND1 iPSC vs EB	2E-6	****
			TBXT iPSC vs EB	3.3E-4	***
			BMP4 iPSC vs EB	3.3E-4	***
			SOX17 iPSC vs EB	2.0E-5	****
			FOXA2 iPSC vs EB	1.9E-4	***
**Appendix Figure S9**					
S9D	Multiple t-tests with post hoc Holm-Sidak method	N/A	***PAX6*** iPSC vs EB	1.7E-4	****
			SOX1 iPSC vs EB	0.0023	***
			HAND1 iPSC vs EB	8.2E-5	****
			TBXT iPSC vs EB	1.7E-4	****
			BMP4 iPSC vs EB	1.4E-4	****
			SOX17 iPSC vs EB	7.8E-5	****

			FOXA2 iPSC vs EB	9.9E-5	****
**Appendix Figure S10**					
S10C	Two-way ANOVA	Sidak's multiple comparisons test	***TNNT2*** WT vs T184K	3.0E-8	****
			MYL2 WT vs T184K	0.0199	*
			MYH7 WT vs T184K	1.0E-5	****
			ACTA1 WT vs T184K	0.0042	**
**Appendix Figure S12**					
S12B	Two-way ANOVA	Sidak's multiple comparisons test	WT vs KI	4.6E-34	****
			KI vs KI treated	6.12E-33	****
S12E	One-way ANOVA	Tukey's multiple comparisons test	WT vs KI	0.0003	***
			KI vs KI treated	0.0037	**
S12F	One-way ANOVA	Tukey's multiple comparisons test	WT vs KI	3.4E-10	****
			KI vs KI treated	5.5E-10	****
S12G (Day sleep)	One-way ANOVA	Tukey's multiple comparisons test	WT vs KI	0.0004	***
			KI vs KI treated	2.0E-07	****

S12G (Day sleep bouth number)	One-way ANOVA	Tukey's multiple comparisons test	WT vs KI	0.0012	**
			KI vs KI treated	0.0348	*
S12G (Day sleep bouth length)	One-way ANOVA	Tukey's multiple comparisons test	WT vs KI	1.5E−10	****
			KI vs KI treated	0.0022	**
S12H (night sleep)	One-way ANOVA	Tukey's multiple comparisons test	WT vs KI	1.8E-09	****
			KI vs KI treated	4.2E-06	****
S12H (night sleep bout number)	One-way ANOVA	Tukey's multiple comparisons test	WT vs KI	4.3E-10	****
			KI vs KI treated	0.0267	*
S12H (night sleep bout length)	One-way ANOVA	Tukey's multiple comparisons test	WT vs KI	4.7E-7	****
			KI vs KI treated	0.0119	*

Appendix Table S2: List of primers used in the study

Primer	Sequence	Purpose
PAX6 Forward	AACGATAACATACCAAGCGTGT	RT-PCR
PAX6 Reverse	GGTCTGCCCGTTCAACATC	
SOX1 Forward	GAGTGGAAGGTCATGTCCGAGG	
SOX1 Reverse	CCTTCTTGAGCAGCGTCTTGGT	
HAND1 Forward	AACTCAAGAAGGCGGATGG	
HAND1 Reverse	GGAGGAAAACCTTCGTGCT	
TBXT Forward	GGTCCAGCCTTGGAATGCCT	
TBXT Reverse	CCGTTGCTCACAGACCACAG	
BMP4 Forward	GCACTGGTCTTGAGTATCCTG	
BMP4 Reverse	TGCTGAGGTTAAAGAGGAAACG	
SOX17 Forward	GTGGACCGCACGGAATTTGA	
SOX17 Reverse	GCTGTCGGGGAGATTCACAC	
FOXA2 Forward	ATGCACTCGGCTTCCAGTATG	
FOXA2 Reverse	TGTTCATGCCGTTCATCCCC	
RNU6-1 Forward	ATTGGAACGATACAGAGAAGATTAG	
RNU6-1 Reverse	AATATGGAACGCTTCACGAAT	
Human *ACTA1* Forward	TCTCACCGACTACCTGATGAA	
Human *ACTA1* Reverse	AGCACAGCTTCTCCTTGATG	
Human *MYH6* Forward	ATATACCTACTCGGGCCTCTT	
Human *MYH6* Reverse	GTCGGAGATGGAGAAGATGTG	
Human *NPPA* Forward	TTGCTGGACCATTTGGAAGA	
Human *NPPA* Reverse	GCTTCTTCATTCGGCTCACT	
Human *NPPB* Forward	TCCTGCTCTTCTTGCATCTG	
Human *NPPB* Reverse	GTAACCCGGACGTTTCCAA	
Human *18s* rRNA Forward	GTAACCCGTTGAACCCCATT	
Human *18s* rRNA Reverse	CCATCCAATCGGTAGTAGCG	
Human *MYH7* Forward	TGAAGGAGGACCAGGTGAT	
Human *MYH7* Reverse	GTAGCGATCCTTGAGGTTGTAG	
Rattus novergicus Myh7 Forward	CCTCGCAATATCAAGGGAAA	
Rattus novergicus Myh7 Reverse	TACAGGTGCATCAGCTCCAG	
Rattus novergicus Acta1 Forward	CCTGGACTTCGAGAATGAGATG	
Rattus novergicus Acta1 Reverse	CGATAAAGGAAGGCTGGAAGAG	

Rattus novergicus Nppb Forward	AGATGATTCTGCTCCTGCTTT	
Rattus novergicus Nppb Reverse	ATCGTGGATTGTTCTGGAGAC	
Rattus novergicus 18s rRNA Forward	GGAACTGAGGCCATGATTAAGA	
Rattus novergicus 18s rRNA Reverse	CAAATGCTTTCGCTCTGGTTC	
Rattus novergicus Nppa Forward	ATTTCAAGAACCTGCTAGACC	
Rattus novergicus Nppa Reverse	TTTTCAAGAGGGCAGATCTAT	
KCNA2 Forward	TCGGTTTTATGAGCTGGGAGAAG	Sanger sequencing
KCNA2 Reverse	ACCTAGAATCTGGAGACCTTTGG	
Sev Forward (From the kit)	GGATCACTAGGTGATATCGAGC	Sendai virus check
Sev Reverse (From the kit)	ACCAGACAAGAGTTTAAGAGATATGT ATC	
KOS transgene Forward (From the kit)	ATGCACCGCTACGACGTGAGCGC	
KOS transgene Reverse (From the kit)	ACCTTGACAATCCTGATGTGG	
GAPDH Forward	GAAGGTGAAGGTCGGAGTC	
GAPDH Reverse	GAAGATGGTGATGGGATTTC	
