# Supplementary figures and images for: KCNA2 variants cause dilated cardiomyopathy, obesity and sleep apnea through RAC-ERK pathway
Source: EMBO Mol Med. 2026 Feb 24;18(4):1134–49. doi: 10.1038/s44321-026-00391-y (PMC13083917; doi:10.1038/s44321-026-00391-y)

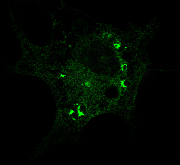

Supplement: Supplementary file 3 — Source data Fig. 2 [file 44321_2026_391_MOESM3_ESM.zip › Figure 2/2D/R189W/R189W_composite .tif (RGB).tif]

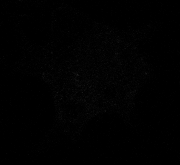

Supplement: Supplementary file 3 — Source data Fig. 2 [file 44321_2026_391_MOESM3_ESM.zip › Figure 2/2D/R189W/R189W_ha_gray RBG.tif]

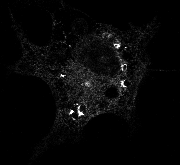

Supplement: Supplementary file 3 — Source data Fig. 2 [file 44321_2026_391_MOESM3_ESM.zip › Figure 2/2D/R189W/R199W_gfp_gray RGB.tif]

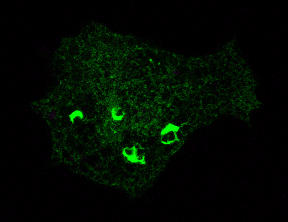

Supplement: Supplementary file 3 — Source data Fig. 2 [file 44321_2026_391_MOESM3_ESM.zip › Figure 2/2D/T184K/T184K_composite .tif (RGB).tif]

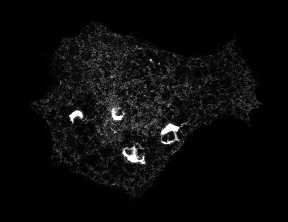

Supplement: Supplementary file 3 — Source data Fig. 2 [file 44321_2026_391_MOESM3_ESM.zip › Figure 2/2D/T184K/T184K_gfp_gray RGB.tif]

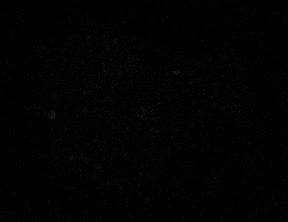

Supplement: Supplementary file 3 — Source data Fig. 2 [file 44321_2026_391_MOESM3_ESM.zip › Figure 2/2D/T184K/T184K_ha_gray RGB.tif]

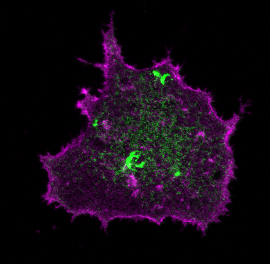

Supplement: Supplementary file 3 — Source data Fig. 2 [file 44321_2026_391_MOESM3_ESM.zip › Figure 2/2D/WT/WT_Composite .tif (RGB).tif]

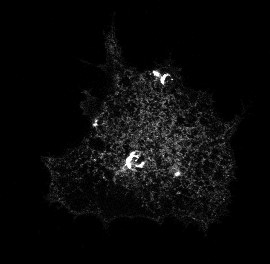

Supplement: Supplementary file 3 — Source data Fig. 2 [file 44321_2026_391_MOESM3_ESM.zip › Figure 2/2D/WT/WT_gfp gray RGB.tif]

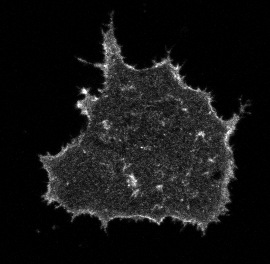

Supplement: Supplementary file 3 — Source data Fig. 2 [file 44321_2026_391_MOESM3_ESM.zip › Figure 2/2D/WT/WT_ha gray RGB.tif]

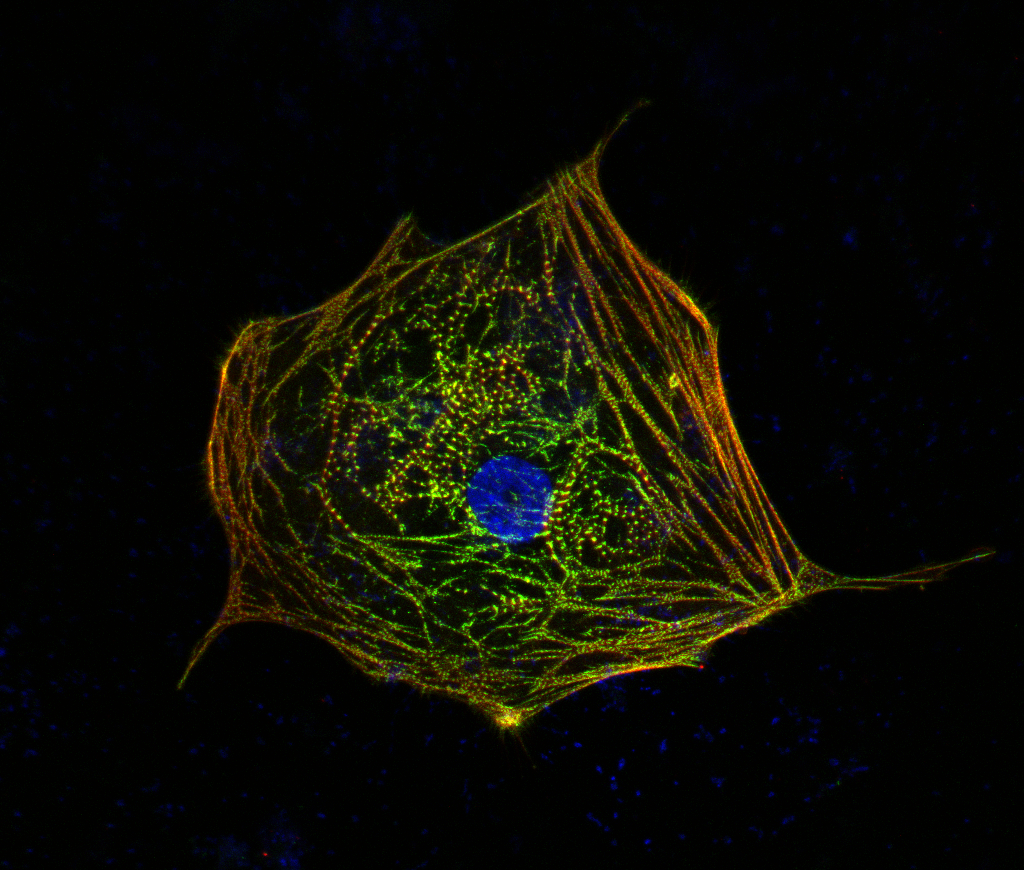

Supplement: Supplementary file 4 — Source data Fig. 3 [file 44321_2026_391_MOESM4_ESM.zip › Figure 3/3A/Cardiomyocytes/T184K/T184K.tif (RGB).tif]

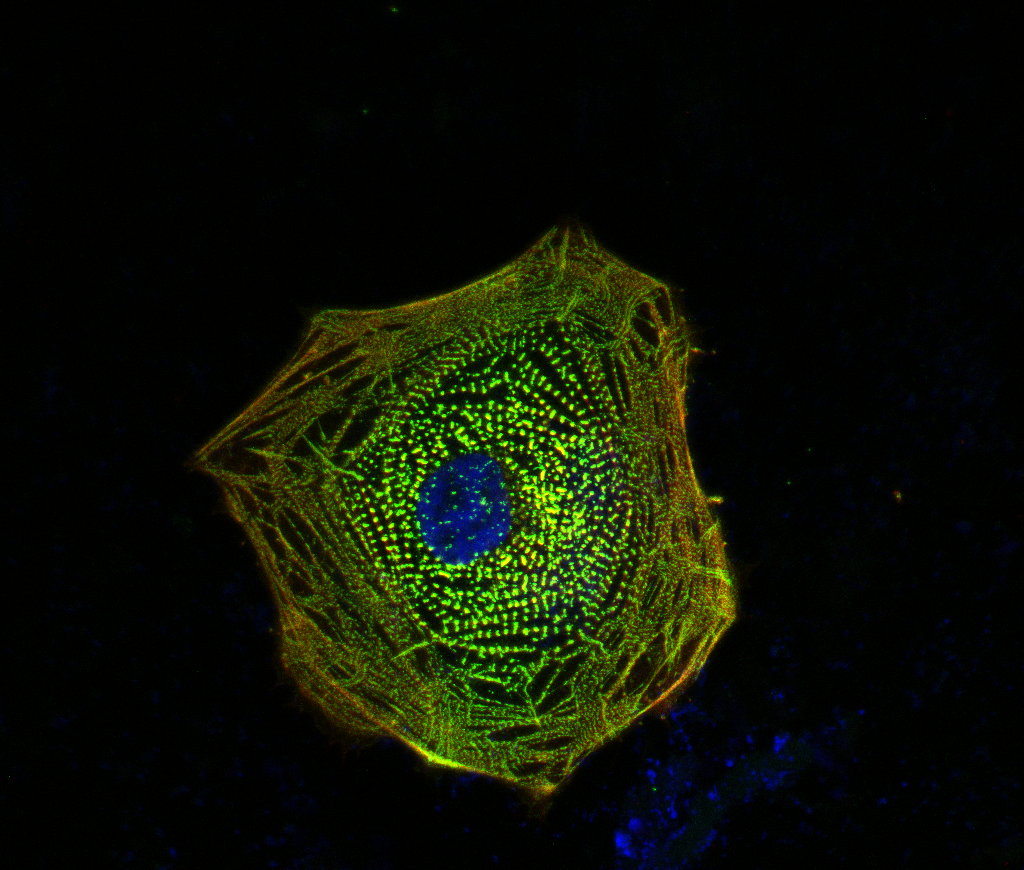

Supplement: Supplementary file 4 — Source data Fig. 3 [file 44321_2026_391_MOESM4_ESM.zip › Figure 3/3A/Cardiomyocytes/T184K_Treated/T184K_Treated.tif (RGB).tif]

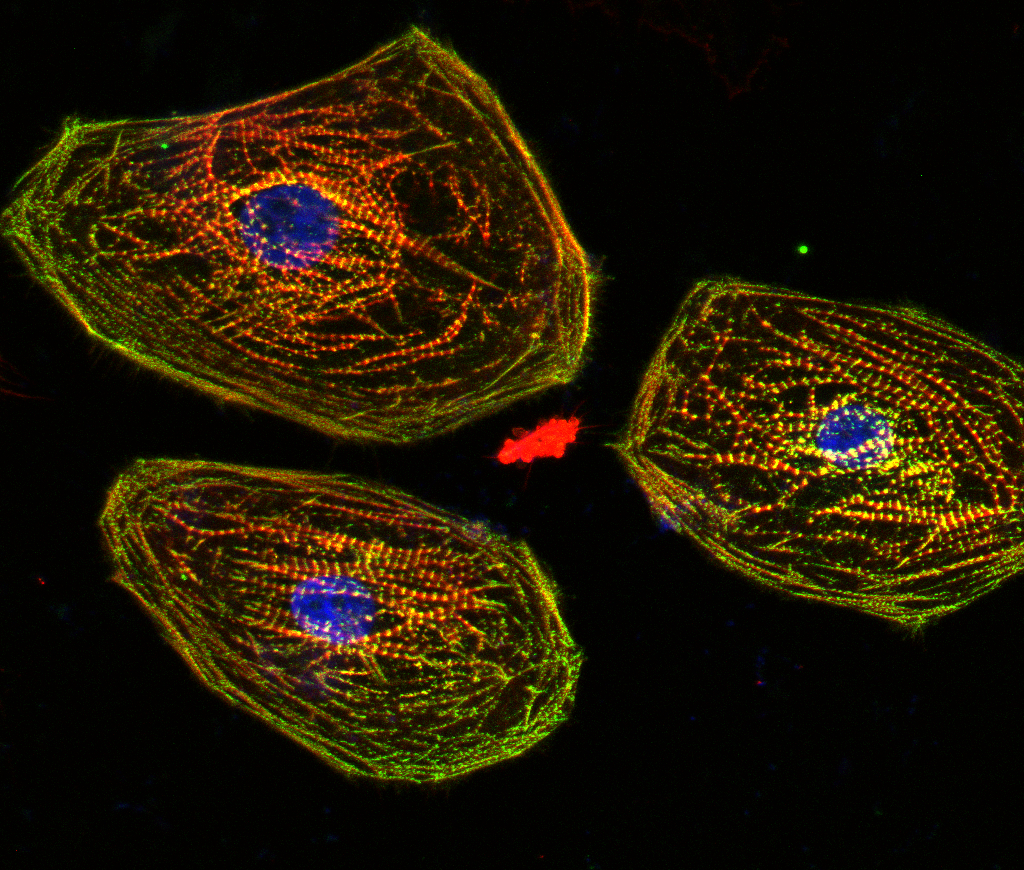

Supplement: Supplementary file 4 — Source data Fig. 3 [file 44321_2026_391_MOESM4_ESM.zip › Figure 3/3A/Cardiomyocytes/WT/WT.tif (RGB).tif]

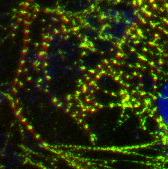

Supplement: Supplementary file 4 — Source data Fig. 3 [file 44321_2026_391_MOESM4_ESM.zip › Figure 3/3A/Sarcomere/T184K/Composite _T184K_sarcomere .tif (RGB).tif]

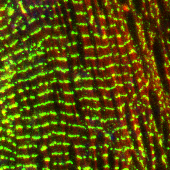

Supplement: Supplementary file 4 — Source data Fig. 3 [file 44321_2026_391_MOESM4_ESM.zip › Figure 3/3A/Sarcomere/T184K_T/Composite T184K_treated_sarcomere.tif (RGB).tif]

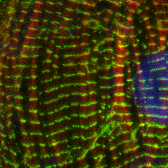

Supplement: Supplementary file 4 — Source data Fig. 3 [file 44321_2026_391_MOESM4_ESM.zip › Figure 3/3A/Sarcomere/WT/Composite_WT_sarcomere .tif (RGB).tif]

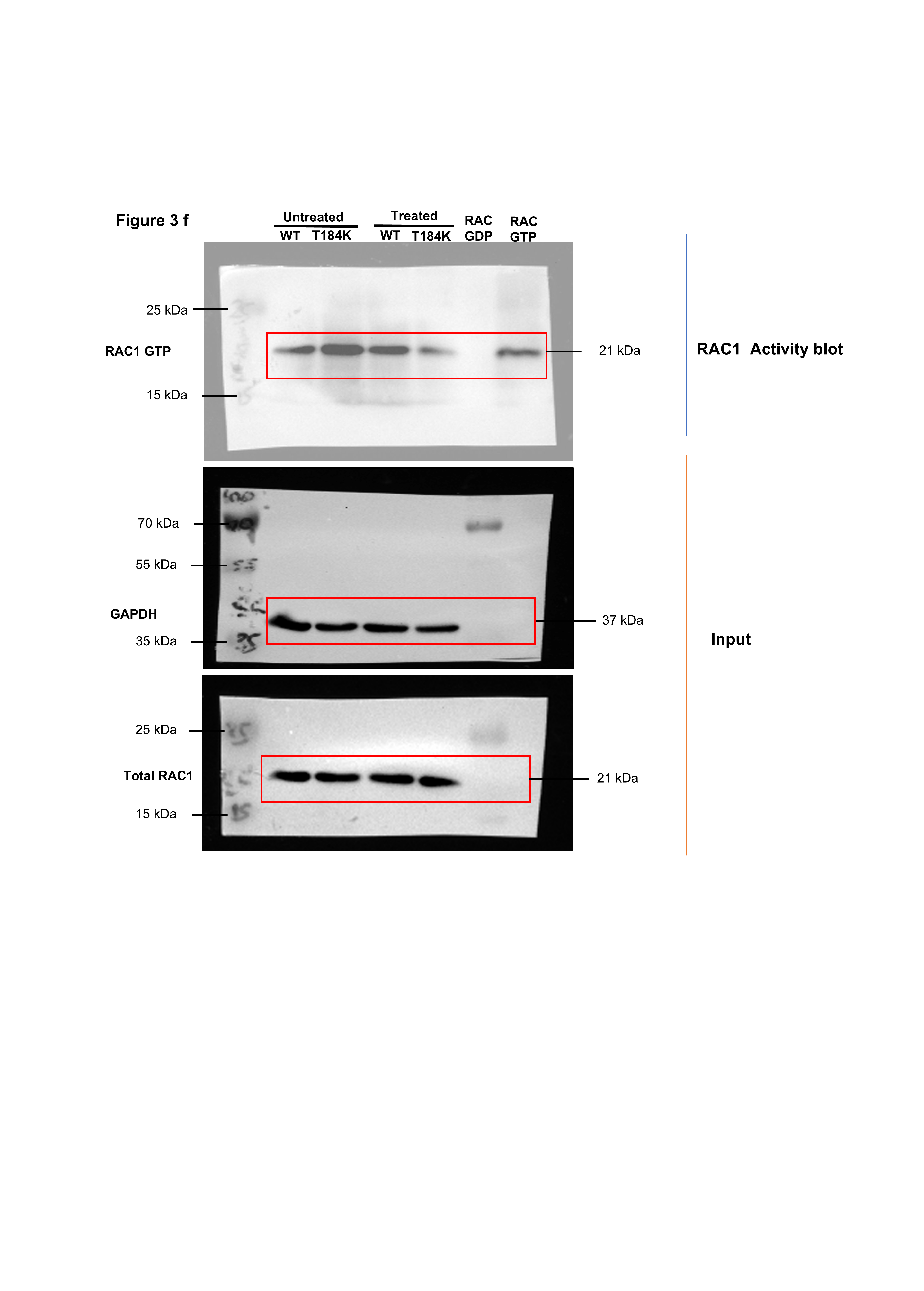

Supplement: Supplementary file 4 — Source data Fig. 3 [file 44321_2026_391_MOESM4_ESM.zip › Figure 3/3F/RAC1 activity full blot.tif]

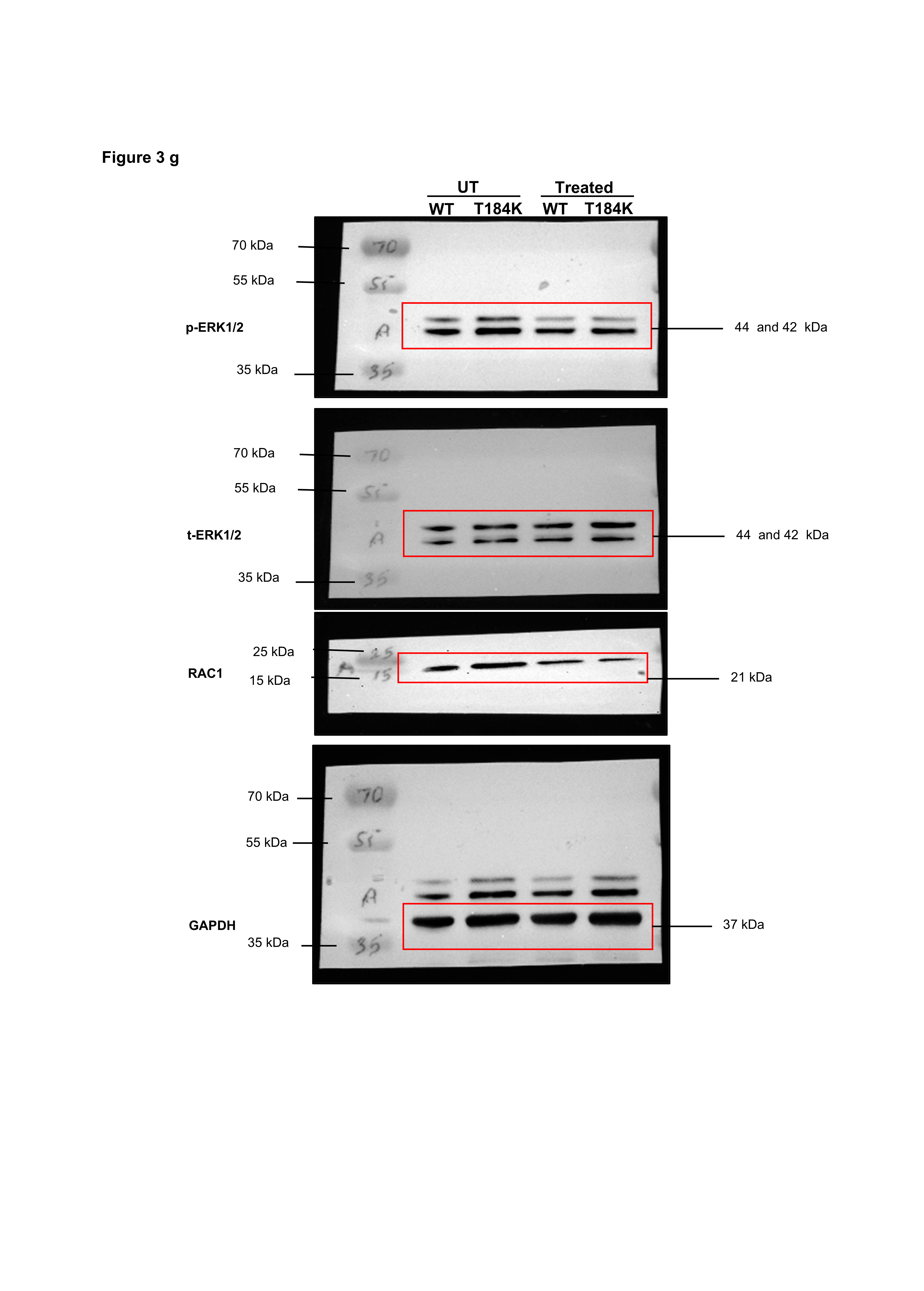

Supplement: Supplementary file 4 — Source data Fig. 3 [file 44321_2026_391_MOESM4_ESM.zip › Figure 3/3G/ERK full blot.tif]

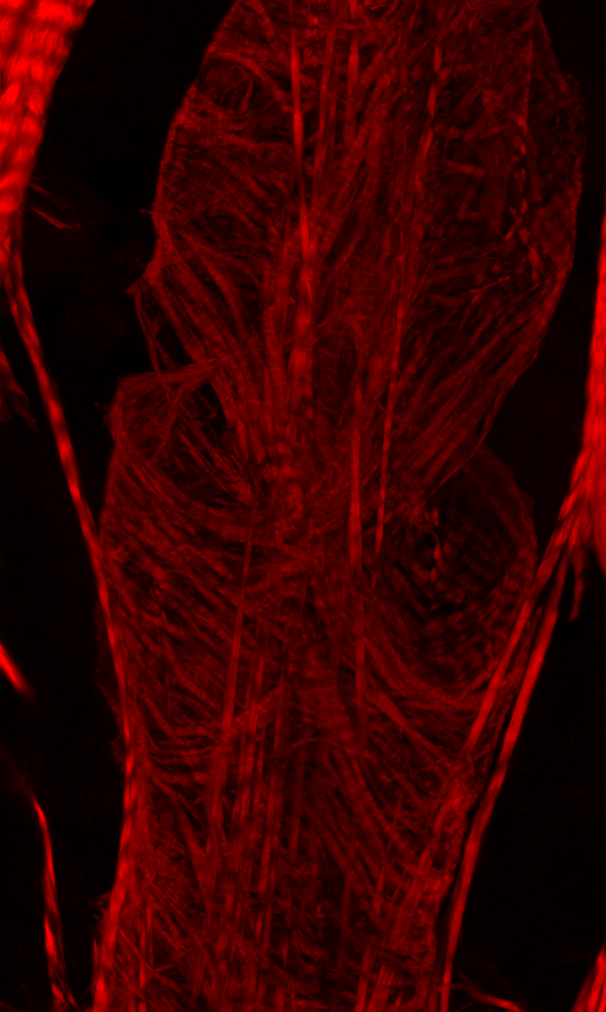

Supplement: Supplementary file 5 — Source data Fig. 4 [file 44321_2026_391_MOESM5_ESM.zip › Figure 4/4A/untreated KI/KI_RGB.tif]

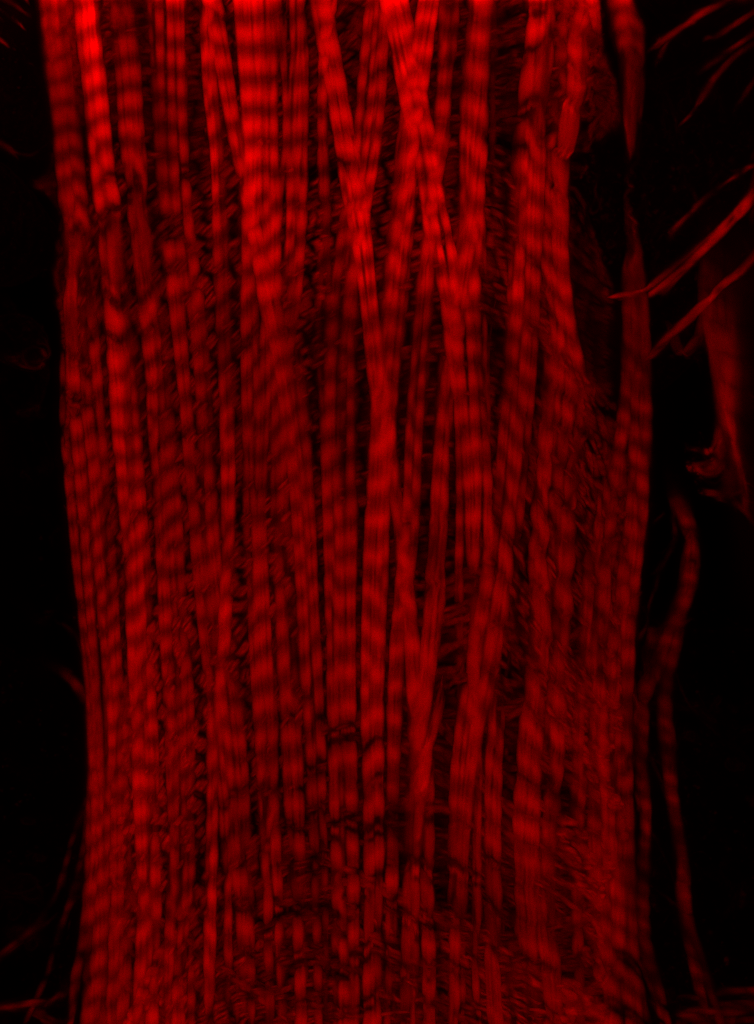

Supplement: Supplementary file 5 — Source data Fig. 4 [file 44321_2026_391_MOESM5_ESM.zip › Figure 4/4A/Untreated wt/WT_RGB.tif]

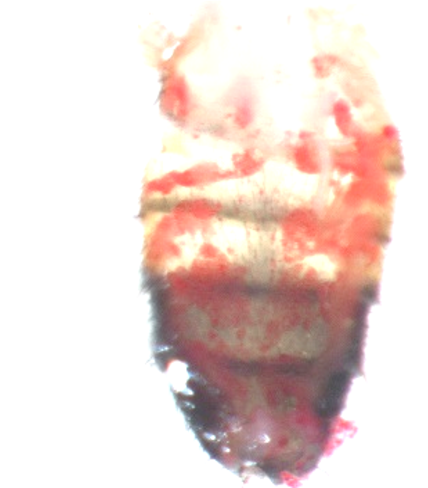

Supplement: Supplementary file 5 — Source data Fig. 4 [file 44321_2026_391_MOESM5_ESM.zip › Figure 4/4C/KI oil o red.tif]

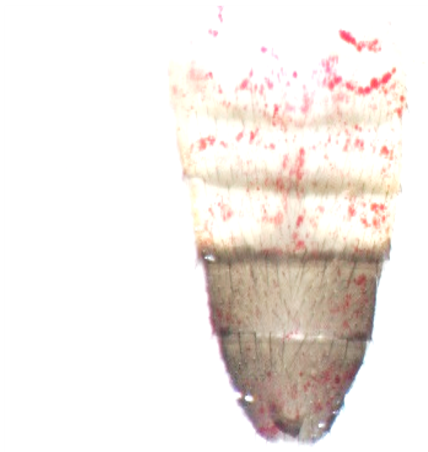

Supplement: Supplementary file 5 — Source data Fig. 4 [file 44321_2026_391_MOESM5_ESM.zip › Figure 4/4C/WT oil o red.tif]

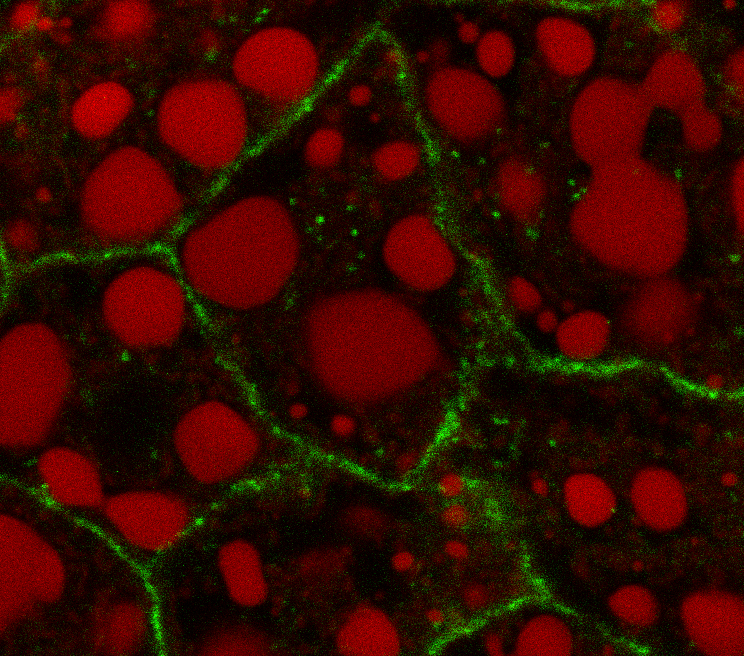

Supplement: Supplementary file 5 — Source data Fig. 4 [file 44321_2026_391_MOESM5_ESM.zip › Figure 4/4D/KI/KI.tif (RGB).tif]

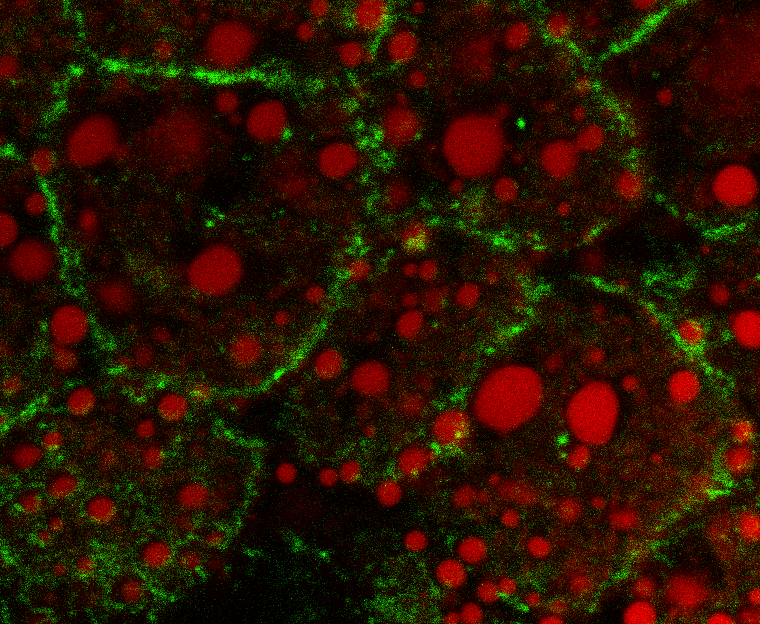

Supplement: Supplementary file 5 — Source data Fig. 4 [file 44321_2026_391_MOESM5_ESM.zip › Figure 4/4D/WT/WT.tif (RGB).tif]
